# Preparation of Viable Human Neurites for Neurobiological and Neurodegeneration Studies

**DOI:** 10.3390/cells13030242

**Published:** 2024-01-27

**Authors:** Markus Brüll, Nils Geese, Ivana Celardo, Michael Laumann, Marcel Leist

**Affiliations:** 1 In Vitro Toxicology and Biomedicine, Department Inaugurated by the Doerenkamp-Zbinden Foundation, University of Konstanz, 78457 Konstanz, Germany; markus.2.bruell@uni-konstanz.de (M.B.); nils.geese@uni-konstanz.de (N.G.); ivana.celardo@uni-konstanz.de (I.C.); 2 Electron Microscopy Centre, University of Konstanz, 78457 Konstanz, Germany; michael.laumann@uni-konstanz.de; 3 Center for Alternatives to Animal Testing in Europe (CAAT-Europe), University of Konstanz, 78457 Konstanz, Germany

**Keywords:** axotomy, Wallerian degeneration, neurite isolation, neurospheres, nicotinamide

## Abstract

Few models allow the study of neurite damage in the human central nervous system. We used here dopaminergic LUHMES neurons to establish a culture system that allows for (i) the observation of highly enriched neurites, (ii) the preparation of the neurite fraction for biochemical studies, and (iii) the measurement of neurite markers and metabolites after axotomy. LUHMES-based spheroids, plated in culture dishes, extended neurites of several thousand µm length, while all somata remained aggregated. These cultures allowed an easy microscopic observation of live or fixed neurites. Neurite-only cultures (NOC) were produced by cutting out the still-aggregated somata. The potential application of such cultures was exemplified by determinations of their protein and RNA contents. For instance, the mitochondrial TOM20 protein was highly abundant, while nuclear histone H3 was absent. Similarly, mitochondrial-encoded RNAs were found at relatively high levels, while the mRNA for a histone or the neuronal nuclear marker NeuN (RBFOX3) were relatively depleted in NOC. Another potential use of NOC is the study of neurite degeneration. For this purpose, an algorithm to quantify neurite integrity was developed. Using this tool, we found that the addition of nicotinamide drastically reduced neurite degeneration. Also, the chelation of Ca^2+^ in NOC delayed the degeneration, while inhibitors of calpains had no effect. Thus, NOC proved to be suitable for biochemical analysis and for studying degeneration processes after a defined cut injury.

## 1. Introduction

There is a dearth of models that enable studies on neurites from central nervous system (CNS) human neurons [1,2]. In this context, a particularly high demand exists for a test system that can be used for a better definition of underlying pathological processes of neurite degeneration (ND) and also can be used to explore experimental and pharmacological intervention options [3,4,5].

Due to their length, neurites demand specialized mechanisms for maintenance and processes that ensure their integrity. This comes with unique cell biological challenges, such as the organization of retrograde and anterograde axonal transport [6], the localized regulation of transcript [7] and protein [8] stability, or the transfer of signals between the cell body and the distant neurite structures [9]. Moreover, their spatial extension renders neurites particularly susceptible to injury. A particular type of damage only found in neurons originates from a separation of the proximal (close to cell body) and distal parts of the neurite. Here, we refer to the separation of cell body and neurite as “axotomy”. This damage can be either caused physically (e.g., crush, compression, or cut), e.g., in the spinal cord or due to a traumatic brain injury, or by a “chemical” block of axonal transport. The latter occurs typically after disturbances of the neuronal cytoskeleton or by protein aggregation within neurites [10,11]. For such reasons, ND often precedes cell death in neurodegenerative diseases [3,12,13,14,15].

It is crucial to continue work on models of CNS neurites to improve our understanding of their homeostatic control and of the pathophysiological processes leading to their degeneration. Several methods have been developed to study neurites as a cellular compartment or to model their injury. These methods have different advantages and drawbacks and include: (i) rodent brain structures (e.g., hippocampus neuropil [16]), (ii) neurites growing into a distinct cell culture compartment in technical devices, such as microfluidic chambers [17,18] or Campenot chambers [19], and (iii) microporous membranes [20,21], outgrowth from plated dorsal root ganglia [22,23,24], or the use of growth restricted spotted cultures [22]. Models for neurite degeneration/regeneration in the CNS include optic nerve cut [25], spinal cord crush injury [26,27], or median forebrain bundle lesioning with 6-hydroxy-dopamine [28,29]. In vitro approaches to study neurite regeneration include, e.g., scratch or wound healing assays [30]. One in vitro approach to study human neurites makes use of neuroblastoma (NTera2)-derived hNT neurons [31,32,33]. Many other studies use SH-SY5Y neuroblastoma cells [34,35]. Some more recent approaches use neurons derived from hNP1 neural precursor cells [36,37]. There is a wealth of mechanistic in vitro studies on neurite pathology in primary rodent neural cultures, but much less information is available for human cells. For instance, some sophisticated systems simultaneously studied axonal and dentritic structures or other detailed compartments [38,39].

The first evidence that ND may be a programmed mode of compartmentalized cell death emerged when a mutant mouse was characterized, in which Wallerian degeneration (the distal degeneration occurring after a nerve cut) was dramatically attenuated [40]. In these “Wallerian degeneration slow” (wld(s)) mice, it was shown that neurite disintegration can be blocked genetically or by pharmacological interventions [41]. In further genetic studies, it was shown that the nicotinamide/NAD^+^ biochemistry is a key factor in controlling neurite death [42,43]. The NAD^+^-degrading enzyme SARM1 is nowadays considered a key executioner [22,44,45,46].

Most knowledge of programmed neurite degeneration was gained from experiments with peripheral neurons [46]. However, CNS diseases are also characterized by progressive neurite degeneration [3,12,47,48]. It has been suggested that processes similar to those in peripheral neurons would control neurite degeneration in neurodegenerative diseases of the CNS, such as Parkinson’s disease or multiple sclerosis [4,46,49].

To further elucidate this, there is a need for a model with the respective neuronal target populations. To model Parkinson’s disease (PD) pathology, dopaminergic central neurons would be best suited. Conditionally immortalized fetal human midbrain neurons (LUHMES) have been characterized and used for such a purpose [50,51]. They have a largely intact genome, allow for the generation of homogeneous, post-mitotic dopaminergic cultures, and have been extensively used to study toxicological effects on neurites [52,53,54]. Moreover, they can form spheroids, which show extensive neurite outgrowth [55,56]. On the basis of these cells, we aimed to set up a novel model to study the biology and pathology of neurites. We planned for this system (a) to display a robust, homogeneous CNS phenotype, (b) to have sufficiently long neurites to allow axotomy, (c) to allow biochemical analyses of neurites, and (d) to show Wallerian-like ND after separation from the cell bodies. The strategy was to use a sequential 3D, then 2D culture (here termed 2.5D). The extensive length of the several mm long neurites and their organized growth allowed the removal of the centrally aggregated cell bodies, i.e., a highly defined axotomy. These cultures were evaluated for suitability for a biochemical analysis of the isolated neurite compartment. Moreover, we developed a method to quantify ND, and to study pharmacological modifiers.

## 2. Materials and Methods

### 2.1. Cell Culture

LUHMES cells were handled as previously described in detail [51,55]. Proliferating LUHMES cells were typically cultivated in poly-L-ornithine hydrobromide (PLO)/fibronectin-coated T75 flasks (Sarstedt, Nümbrecht, Germany) in 10 mL of proliferation medium. The proliferation medium consisted of advanced Dulbecco’s modified eagle medium (DMEM)/F12 (Gibco, Billings, MT, USA) supplemented with 2 mM glutamine (Sigma-Aldrich, St. Louis, MO, USA), 1× N2 supplement (Gibco, USA), and 40 ng/mL Fibroblast growth factor 2 (FGF2, R&D Systems, Minneapolis, MN, USA). Cells were passaged into a new flask every 2–3 days when they reached approximately 80% confluence. To passage the cells, the medium was removed and the cells were detached by incubation in 2 mL 0.05% trypsin–EDTA solution (Gibco, USA) for 3 min at 37 °C. The trypsinized cell suspension was added to 8 mL advanced DMEM/F12 without supplements. The cell suspension was centrifuged at 300× *g* for 5 min (1200 rpm, Heraeus Multifuge 1 S-R, ThermoScientific, Waltham, MA, USA) to wash out the trypsin. The supernatant was removed and the pellet was resuspended in 1 mL of advanced DMEM/F12. The cell number was determined by counting in a Neubauer counting chamber. Cells were seeded at a confluency of 40,000 cells/cm^2^ for two days of proliferation and 13,000 cells/cm^2^ for three days of proliferation. Cells were cultivated at 37 °C with 5% CO_2_. To initiate differentiation, proliferating LUHMES cells were seeded at a confluency of 120,000 cells/cm^2^ in differentiation medium in T75 flasks (displayed as day of differentiation (DoD) 0 in figures). The differentiation medium contained advanced DMEM/F12 supplemented with 2 mM glutamine, 1 mM dibutyryl-cyclic adenosine monophosphate (db-cAMP) (Sigma-Aldrich, USA), 2 ng/mL glial derived neurotrophic factor (GDNF) (R&D Systems, USA), and 2.25 μM tetracycline (Sigma-Aldrich, USA). LUHMES cells were differentiated for two days before spheroid generation. Red fluorescent protein (RFP)-expressing LUHMES (characterized in [57]) were cultivated the same way. The LUHMES cell line (ATCC: CRL-2927) was genetically characterized in [50]. Annotated variant files for the LUHMES cell line (UKN Subpopulation) have been deposited for public access at ELIXIR Luxembourg (accession number: 10.17881/LCSB.20180321.01).

### 2.2. Coating

For maintenance culture and differentiation, LUHMES cells were cultured in T75 flasks (Sarstedt, Germany) coated with 43 μg/mL PLO (Sigma-Aldrich, USA) and 1 μg/mL fibronectin (Sigma-Aldrich, USA) in milliQ H_2_O. Flasks were incubated with 10 mL coating solution at 37 °C overnight. After incubation, the coating solution was aspirated and the flasks were washed twice with milliQ H_2_O. If not used immediately, the coated flasks were dried and stored at 4 °C. For spheroid generation and culture, plates were coated with BIOFLOAT™ FLEX coating solution (faCellitate, Mannheim, Germany). To this end, untreated round bottom 96 well plates (Corning 3795) were incubated for 2 min at room temperature (RT) with the ultra-low attachment coating solution. The coating solution was aspirated and plates were left to dry under a laminar flow hood for at least 1 h. Plates were stored for up to 4 weeks at 4 °C.

### 2.3. Spheroid Generation and Plating

To generate LUHMES spheroids, 10,000 DoD 2 LUHMES cells were seeded in 100 μL differentiation medium per well in ultra-low attachment round-bottom 96-well plates. To accumulate the cells at the well bottom, plates were centrifuged at 300× *g* (Heraeus Multifuge 1 S-R, ThermoScientific, USA) for 5 min. Plates were incubated at 37 °C at 5% CO_2_. A half medium exchange was performed on DoD 6 or 7. For spheroid plating, 50 µL of a suspension of 5% Matrigel (*v*/*v*) in cold differentiation medium was added to 96-well cell culture plates and then incubated for at least 30 min at 37 °C. To transfer LUHMES spheroids to flat bottom plates, DoD9 spheroids were then taken up in 50 μL medium with cut-off microliter pipette tips and separately seeded onto Matrigel (1 organoid/well), which resulted in a Matrigel concentration of 2.5% in the well. Plates were gently rocked to ensure the central placement of the organoid in the well. Plated organoids were incubated at 37 °C with 5% CO_2_ [55].

### 2.4. Neurite Isolation

In order to induce axotomy or to isolate neurites, cell bodies were removed from plated LUHMES spheroids (2.5D). Spheroids were cultured for neurite outgrowth on Matrigel for 5 or 6 days. The central somata dome was manually removed under visual control with a 1000 µL (blue) pipette tip (E1001, Neolab, Heidelberg, Germany). To ensure clean separation of neurites from somata, the pipette tip was vertically oriented in the light path of a light microscope (Figure S2). The cut was induced by applying pressure on the neurites, resulting in a circular axotomy. The central spheroid was removed with the pipette tip, according to the experimental needs (e.g., for the biochemical analysis of the neurites).

### 2.5. Ribonucleic Acid (RNA) Isolation, Reverse Transcription and Real-Time Quantitative Polymerase Chain Reaction (RT-qPCR)

For RNA isolation, 30 wells were pooled per sample. Each well was sampled with 25 µL TRIzol reagent (PeqGOLD Trifast™ (VWR, Radnor, PA, USA)), resulting in a total volume of 750 µL. RNA was isolated according to the manufacturer’s instructions. To increase the RNA yield, 1 µL of GlycoBlue coprecipitant (Thermo Fisher, Waltham, MA, USA) was added to each sample before phase separation. The resulting RNA pellet was resuspended in 10 µL RNAse-free water (Gibco, USA). The RNA concentration and purity was determined by absorbance measurement with a Nanodrop. Reverse transcription was performed according to the manufacturer’s instructions with 500 ng total RNA, using the i-Script™ Reverse Transcription Supermix (Bio-Rad™, Hercules, CA, USA). Quantitative real-time PCR was performed with the SsoFast™ EvaGreen^®^ Supermix (Bio-Rad™, CA, USA) according to the manufacturer’s instructions, using the real-time PCR Detection System Rotor-Gene Q (Qiagen, Venlo, The Netherlands) for the determination of the cycle threshold (Ct) values. Genes with a Ct > 40 were considered as “not expressed”. Ct values were normalized to the geometric mean of the expression of two housekeeping genes (ACTB and RPL13A, ΔCt). To evaluate the relative abundance of transcripts, we calculated the fold difference between ΔCt of the transcripts of interest between NOC and whole cell samples (ΔΔCt). A list of used primers can be found in Supplementary Table S1.

### 2.6. Western Blots

For the Western blot analysis of neurite proteins, 10 wells of isolated neurites or plated spheroids per sample were lysed in 10 µL 1× Laemmli buffer per well, pooled together, and heated for 5 min at 95 °C. Lysates were centrifuged for 1 min at 10,000× *g* through NucleoSpin Filters (Macherey-Nagel GmbH, Duren, Germany) to break down long DNA strands in uncut samples. Twenty microliters of lysates were loaded onto 10% SDS gels and gels were run for 30 min at 80 V and then at 120 V until bands reached the bottom of the gel. Proteins were transferred onto nitrocellulose membranes (Amersham, UK) using the iBlot™ 2 dry blotting system (Invitrogen, Waltham, MA, USA). Membranes were blocked with 5% bovine serum albumin (BSA, *w*/*v*) in Tris buffered saline (TBS)-Tween (0.5% (*v*/*v*)) overnight at 4 °C. Respective primary antibodies were incubated at 4 °C overnight. Membranes were washed three times with 5% BSA (*w*/*v*) in TBS-Tween (0.5% (*v*/*v*)) at RT for 10 min, and then incubated with horseradish peroxidase-conjugated secondary antibodies for 1 h at RT. For visualization, ECL Western blotting substrate (Pierce/Thermo Fisher Scientific, Rockford, IL, USA) was used and imaged with a Fusion-SL 3500 WL device with Fusion software (Version 15.18, Bio-Rad™, Hercules, CA, USA). A list of used antibodies can be found in Supplementary Table S2.

### 2.7. Immunocytochemistry

For immunocytochemistry, spheroids were plated on chambered polymer coverslips (ibidi 80826, Gräfelfing, Germany). Isolated neurites were fixed by replacing half the medium with 10% neutral buffered formalin (Leica Biosystems Richmond, Inc., Richmond, IL, USA) and incubated for 30 min at RT. The fixation solution was removed and the samples were washed once with Dulbecco’s phosphate-buffered saline (DPBS, Thermo Fisher Scientific, Waltham, MA, USA), blocked for 1 h at RT, and permeabilized with 0.1% Triton^®^-X100 and 5% fetal bovine serum (FBS) in DPBS (blocking buffer). Incubation with the respective primary antibodies in blocking buffer was performed at 4 °C overnight. After this, a washing step was performed twice with blocking buffer. Then, incubation with respective secondary antibodies diluted in blocking buffer was performed for 1 h at RT. After washing twice in DPBS, samples were covered with Aqua-Poly/Mount (Polyscience, Warrington, PA, USA) and left for drying until imaging. Cells stained with immunofluorescent antibodies were imaged using a Zeiss AxioObserver epifluorescence microscope with ZEN 2 pro blue edition software (Zeiss, Oberkochen, Germany). A list of used antibodies can be found in Supplementary Table S2.

### 2.8. Scanning Electron Microscopy

To prepare scanning electron microscopy (SEM) samples, 12 mm glass coverslips were pre-coated with 86 μg/mL PLO (Sigma-Aldrich, USA) in milliQ H_2_O in 24-well plates for 48 h at 37 °C. Coverslips were washed once with milliQ H_2_O. Coverslips were then coated with a cold 5% Matrigel suspension in DMEM/F12. The coverslips were incubated for 30 min at 37 °C. Afterwards, the Matrigel suspension was removed and replaced with 450 µL warm differentiation medium (referred to as “minimal Matrigel coating”). LUHMES spheroids were transferred onto coverslips on DoD 9 in 50 µL medium (500 µL medium in total) and left for neurite outgrowth until DoD 14. For pre-fixation, half the medium was replaced by 3% formaldehyde and 2% glutaraldehyde in 0.1 M 4-(2-hydroxyethyl)-1-piperazineethanesulfonic acid (HEPES) buffer with 0.09 M sucrose, 0.1 M MgCl_2_, and 0.1 M CaCl_2_ at pH 7.0 for 15 min at room temperature. Subsequent chemical fixation was performed at 4 °C unless otherwise noted; the fixation of spheroids was undertaken in the above-noted pre-chilled fixative for 30 min. Samples were washed three times in pre-chilled 0.1 M HEPES buffer with 0.09 M sucrose, 0.1 M MgCl_2_, and 0.1 M CaCl_2_ at pH 7.0 for 5 min each. Then, samples were passed through graded concentrations of pre-chilled 30% and 50% ethanol for 5 min each and of pre-chilled 70% ethanol for 7 min. Dehydration was continued at room temperature in 10% steps of 10 min each and finished in ethanol over a molecular sieve for 10 min twice. Samples were critical-point-dried in CO_2_ in a Leica EM CPD300 (Leica Microsystems, Wetzlar, Germany) and sputter coated with a 6 nm thick layer of platinum in a Quorum Q150R ES (Quorum technologies, Laughton, UK). Micrographs were produced using a Zeiss Auriga FESEM (Zeiss, Oberkochen, Germany), and large area imaging of whole spheroids (Supplementary Figure S1) was carried out with Atlas 5 (Zeiss, Oberkochen, Germany).

### 2.9. Measurement and Quantification of Degeneration

For the measurement of neurite degeneration, neurites were incubated with calcein- acetoxymethylester (3.2 µM) for 1 h at 37 °C, or RFP-expressing LUHMES were used. Neurites were imaged with a Zeiss AxioObserver epifluorescence microscope with a 20× objective (Plan-Neofluar, NA = 0.4). Fields were selected manually. For each condition, 10 images were recorded at a resolution of 1376 × 1104 px. To evaluate the degeneration parameters “fragmentation” and “integrity”, images were processed in FIJI/ImageJ (Version 1.57q). Images were background-corrected (subtract background pixel radius = 50 px), and an unsharp mask (strength = 0.6, radius = 2 px) and a median filter were applied (radius = 1 px). The processed images were then automatically thresholded (method = mean) to binary images. Binary images were analyzed with the “analyze particles” function. A lower threshold for object size was set to 10 µm^2^. The count of objects (*count_sample_*) and their average size (*size_sample_*) in sample images was evaluated. Fragmentation was calculated from the object count, relative to fully degenerated neurites (*count_cut_*). Neurites recorded under control conditions at >18 h after the cut were considered to be fully fragmented and, therefore, were set as 100% for normalization. In addition to this upper limit normalization, fragmentation data were baseline corrected: the object count in images of intact neurites (*count_intact_*) was set to a 0% fragmentation value. Images of intact neurites were obtained from intact 2.5D samples, or from NOC isolated immediately before measurement.

$$Fragmentation\;\left[\%\right]=\frac{count_{sample}-count_{intact}}{count_{cut}-count_{intact}}*100\%$$

The integrity of sample images was calculated from the average object size (*size_sample_*) relative to intact neurites. The average sizes of objects in images of intact neurites (*size_intact_*) were used for normalization and set to 100% integrity. Images of intact neurites were obtained from intact 2.5D samples, or from NOC isolated immediately before measurement. The average object size of neurites at >18 h after the cut were used as baseline corrections and were set to 0% integrity (*size_cut_*). For variance stabilization, values were logarithmized.

$$Integrity\;\left[\%\;of\;intact\right]=\frac{\log\left(size_{sample}\right)-\log\left(size_{cut}\right)}{\log\left(size_{intact}\right)-\log\left(size_{cut}\right)}*100\%$$

### 2.10. Adenosine Triphosphate (ATP) Assay

Neurite ATP was measured with the CellTiter-Glo™ 2.0 assay (G9061, Promega, Madison, WI, USA), according to the manufacturer’s instructions. Briefly, CellTiterGlo assay mix was diluted in equal parts with PBS containing 0.5% Triton-X100. After neurite isolation, 50 µL reaction mix was added to the cell culture medium and incubated for 5 min at RT on a shaker. Then, 100 µL was transferred to a white assay 96-well plate. A dilution of ATP was used for assay calibration (highest concentration: 10 µM). Luminescence was measured using a plate reader (Infinite M200Pro, Tecan, Morrisville, NC, USA).

### 2.11. Nicotinamide Adenine Dinucleotide (NAD(H)) Assay

The total pool of NAD+ and NADH in neurites was measured with the NAD/NADH-Glo™ assay (G9241, Promega, USA), according to the manufacturer’s instructions. Briefly, the medium of isolated neurites was removed and replaced with 25 µL DPBS. To each well, 25 µL of the reaction mix was added. Neurites were lysed by pipetting up and down. Then, 40 µL was transferred to a white assay 96-well plate. A dilution of NAD+ was used for assay calibration (highest concentration: 500 nM). Luminescence was measured every 5 min using a plate reader (Infinite M200Pro, Tecan, Morrisville, NC, USA). The strongest signal which was still in the linear range was used for analysis.

### 2.12. Statistical Analysis

Statistical analysis was performed with GraphPad Prism 7 software. Biological replicates are indicated by “N” and technical replicates are indicated by “n” in the figures or figure legends. Experiments were conducted in at least three independent biological replicates, except when stated otherwise. Statistical tests and significance levels are indicated in the figure legends. *p*-values < 0.05 were regarded as statistically significant.

## 3. Results and Discussion

### 3.1. Preparation of Organized Neuronal Cultures with Distinct Neurite-Only Areas

**Figure 1:**
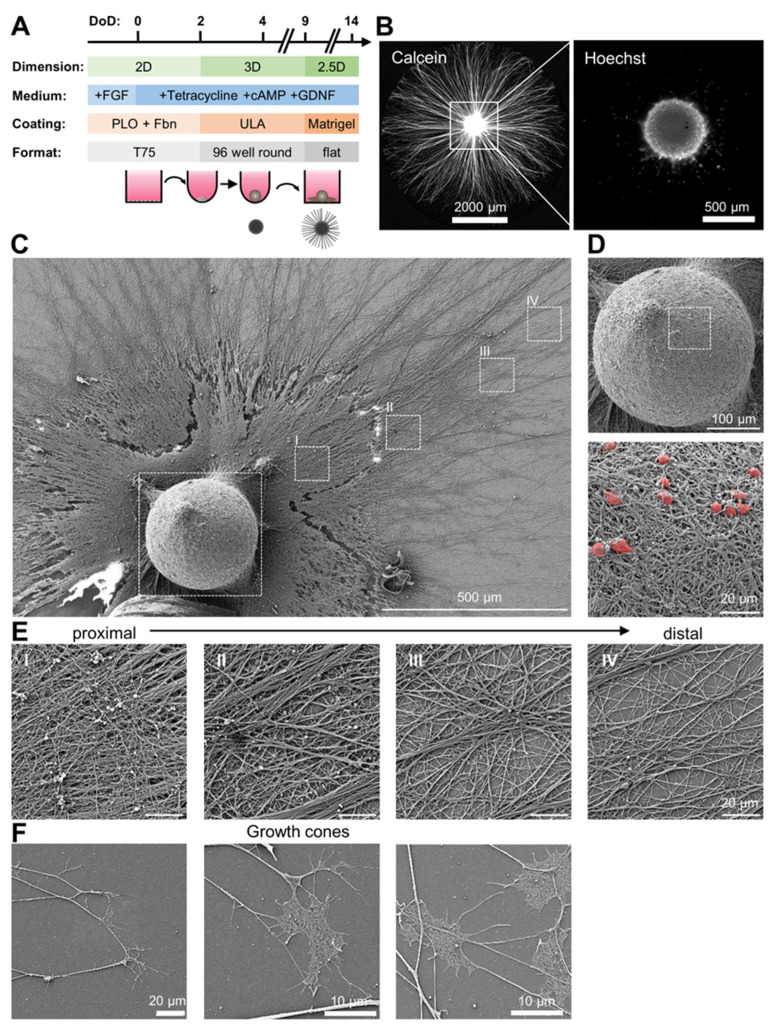
Spatial separation of neurites and cell bodies in 2.5D culture of LUHMES spheroids. (**A**) Schematic overview of culture protocol to obtain plated LUHMES spheroids. LUHMES spheroids were generated in round bottom plates by seeding 10,000 cells per well on day of differentiation (DoD) 2. Spheroids were transferred to a Matrigel-coated flat bottom plate on DoD 9, where they grew out neurites radially. Spheroids were left to grow neurites for 5 days (until DoD 14). FGF = fibroblast growth factor, cAMP = cyclic adenosine monophosphate, GDNF = glial cell-derived neurotrophic factor, Fbn = fibronectin, PLO = poly-L-ornithine, ULA = ultra-low attachment plastic surface, T75 = tissue culture flasks. (**B**) Representative image of a calcein-AM/Hoechst (H-33342)-stained plated spheroid after 5 days of neurite outgrowth (DoD 14). The central cell bodies are shown as the image insert on the right (DNA stained with H-33342). Note that the nuclear stain is shown at a higher magnification and a different focal plane than the whole organoid in the calcein channel. The image was focused on the outer nuclei. (**C**) Scanning electron microscopy (SEM) image of a plated spheroid 5 days after plating (DoD 14) on “minimal Matrigel coating”. White open squares indicate images shown in detail in (**D**,**E**). Note that higher Matrigel amounts were used in standard cultures (see Figure S1), but “minimal Matrigel conditions” were used to obtain cleaner SEM images. (**D**) Higher magnification image of the central spheroid. A detailed image of the spheroid surface is shown below. Cell bodies were pseudo-colored in red. (**E**) Higher magnification images of the neurites radially grown out of the spheroid. Note the occurrence of neurite blebs (2–3 µm in diameter) on proximal, but not distal, neurites. (**F**) Example SEM images of growth cones present at the distal tips of the outgrown neurites.

We generated a neuronal growth pattern in a culture dish in which large areas contained only neurites. These regions of interest were spatially separated from the clearly identifiable area that contained cell bodies. For this purpose, we used a sequential protocol of 3D and 2D cultures. LUHMES spheroids formed spontaneously in ultra-low adherence dishes, as described previously [55]. After plating on Matrigel, the somata remained in place, while neurites extended radially, until they reached the well walls after about 5 days (Figure 1A). At this time, they reached a length of up to 3 mm (distance from cell bodies to neurite tips). In general, a single spheroid covered one whole well, of a 96-well plate, with neurites (Figure 1B). Using scanning electron microscopy, structural details of this culture system were explored (Figure 1C–F). We found that the original spheroid structure remained as high dome, where it had been plated. This 3D structure contained cell bodies, embedded in a very dense coil of neurites. The domes were surround by a dense, mostly 2D-organized neurite network. This network became sparser and increasingly flattened with increasing distance to the center (Figure 1E). At the very distal end, growth cones were readily observed. These extremely flattened structures indicated a still-ongoing neurite outgrowth (Figure 1F). Because of the combination of 3D structures (central dome) with a mostly 2D-organized neurite network, we called this culture format 2.5D. This system allows for various microscopic observations of neurite structure or function without disturbance or interference from cell bodies.

### 3.2. Neurite-Only Cultures (NOC)

**Figure 2:**
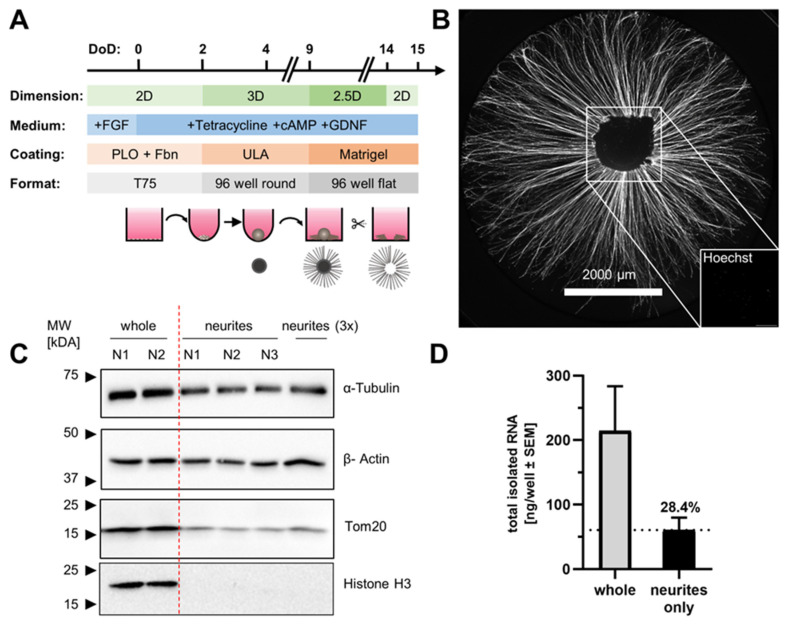
Production and characterization of neurite-only cultures (NOC). (**A**) Schematic overview of the process to generate neurite-only cultures (NOC) from plated LUHMES spheroids. Five days after plating (DoD 14), the central spheroids (containing all somata) were manually removed from the culture. The resulting NOC were either analyzed directly after soma removal, or at different later time points. FGF = fibroblast growth factor, cAMP = cyclic adenosine monophosphate, GDNF = glial cell-derived neurotrophic factor, Fbn = fibronectin, PLO = poly-L-ornithine, ULA = ultra-low attachment plastic surface, T75 = tissue culture flasks. (**B**) Representative image of isolated neurites. Note the absence of any nuclei in the image insert (same scale). N.B.: the non-cut counterpart is shown in Figure 1B; a representative overview across many wells is provided in Figure S2. (**C**) Western blot analysis of marker proteins. To obtain protein samples, 10 wells of plated spheroids with cell bodies (whole) or without cell bodies (neurites) were pooled for each sample. Each well was sampled with 20 µL Laemmli buffer. The “3×” sample contains neurites sampled with 7µL Laemmli buffer per well to obtain higher protein concentrations (30 wells were pooled in total). The same volume of sample (20 µL) was loaded in each lane. (**D**) The total RNA was prepared from neurites or whole cell samples. For each biological replicate, 30 wells were pooled and the total amount of RNA per sample was quantified by UV spectroscopy (N = 5).

The above-described 2.5D cultures were utilized to produce neurite-only cultures (NOC). To this end, plated LUHMES spheroids were left to grow neurites for 5 days (until DoD 14). Then, the central somata dome was cut out (Figure 2A, Figure S2A). This “circular axotomy” resulted in cell culture dishes containing a vast network of radially oriented neurites, but without any somata (Figure 2B). The procedure was performed under microscopic control with high precision. A skilled operator prepared 60 wells within about 15 min, and about two thirds of the originally plated spheroids resulted in high quality NOC (Figure S2B,C). Analysis of the NOC proteins revealed that there was an abundance of cytoskeletal proteins (α-tubulin and β-actin) and, e.g., a mitochondrial protein (Tom20); however, we could not detect the nuclear protein Histone H3 (Figure 2C). This absence of nuclear proteins and the failure to detect nuclei by fluorescence microscopy indicated that the NOC generated by this method were indeed free of cell somata.

The possibility of analyzing proteins from the NOC opens up new possibilities of investigating protein distribution in neurons. We also explored whether RNA could be prepared from NOC. The pooling of multiple wells enabled the purification of neurite RNA. The average yield was ca. 60 ng RNA per well of NOC. This amount corresponded to 28.4 ± 8.7% of the RNA that could be isolated from intact 2.5D cultures (Figure 2D). These findings suggest that about one quarter to one third of the total cellular RNA was located within the neurites.

### 3.3. Quantitative Analysis of Neurite RNA

**Figure 3:**
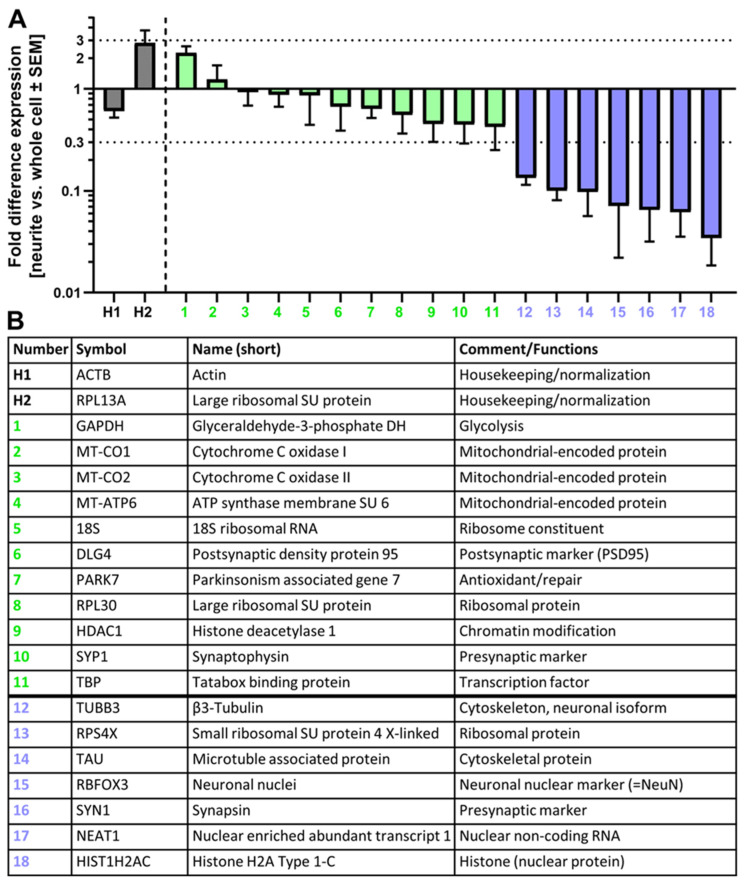
Quantification of relative RNA abundances in neurites. (**A**) RNA was isolated from NOCs or whole cell preparations. The same amount of total RNA was used for analysis by real-time quantitative PCR (RT-qPCR). Expression data were normalized to the geometric mean of ACTB and RPL13A levels (housekeeping genes). The ratio of normalized RNA levels of NOCs vs. whole cell samples was calculated and displayed. Neurite RNAs which were at least 3-fold less abundant (dotted line) in neurites are depicted in blue, and RNAs which were found at similar abundance (fold difference < 3) are depicted in green. Housekeeping genes are shown in grey. Detailed data are disclosed in Figure S3. (**B**) Information on the RNAs shown in (**A**). Where applicable, the names of the translated proteins are shown. DH = dehydrogenase, SU = subunit.

We performed a pilot study to determine whether individual RNAs may be quantified in NOC. The transcripts for a selection of 20 RNA species were analyzed by real-time quantitative PCR. The normalization of RNAs in a subcellular compartment is a non-trivial issue, and the best method depends on the question studied. In our feasibility study, we chose to look only at abundancy ratios between intact 2.5D cultures and NOC. This allowed us to ask the question of whether all RNAs showed the same ratio, or whether some differences were observable. In the first step, we normalized the expression levels to two commonly used housekeeping genes (ACTB and RPL13A), which were highly expressed in both whole cell and neurite-only samples. In the second step, we compared relative expression levels found in neurites to the relative expression in whole 2.5D preparations. Some transcripts were expressed at a similar relative level (±3 fold) in neurites and whole cell samples. A subset of mRNA was about 10× less abundant in neurites compared to whole cell samples (Figure 3A). When judging such a depletion ratio, it is important to consider that we did not compare somata to neurites, but rather complete cultures (somata + neurites) to NOC. Thus a ratio of 10 may mean a very strong depletion in neurites relative to somata. Genes which we found to be expressed at relatively high levels in NOC included mitochondrially expressed genes (MT-CO1, MT-CO2, and MT-ATP6). If one assumes that the mRNA is localized within mitochondria, and that mitochondria are present in both somata and neurites, this result is as it would be expected. It also indicates (though it does not prove) that our method of ratio comparison delivers some meaningful output. One may consider, in future experiments, using such mitochondrial mRNAs as housekeeping normalization standards. We also detected ribosomal RNA (18S) at high levels in NOC. This indicates the presence of ribosomes in neurites. Recently ribosomes have indeed been detected in neurites of the CNS [58,59,60]. Some of the transcripts we found to be depleted in neurites were genes encoding for nuclear proteins (e.g., HIST1H2C and RBFOX3) (Figure 3B). Also, the non-coding RNA NEAT1 was not detectable in NOC but was readily expressed in whole cell preparations (Figure S3). Since NEAT1 is known to be localized in nuclei, these data indicated the absence of nuclei in NOC.

Some transcripts appeared to be partially depleted in neurites, but they still had sizable levels compared to the chosen housekeeping genes (e.g., TUBB3) (Figure S3). Overall, these pilot studies exemplify the fact that RNA can be isolated from NOC and analyzed by qPCR. This allows for the investigation of neuronal RNA distribution and may be used as a tool to investigate the transport, regulation, maintenance, and local translation of neurite RNA in the future.

### 3.4. Time Course of Axotomy-Induced Neurite Degeneration

**Figure 4:**
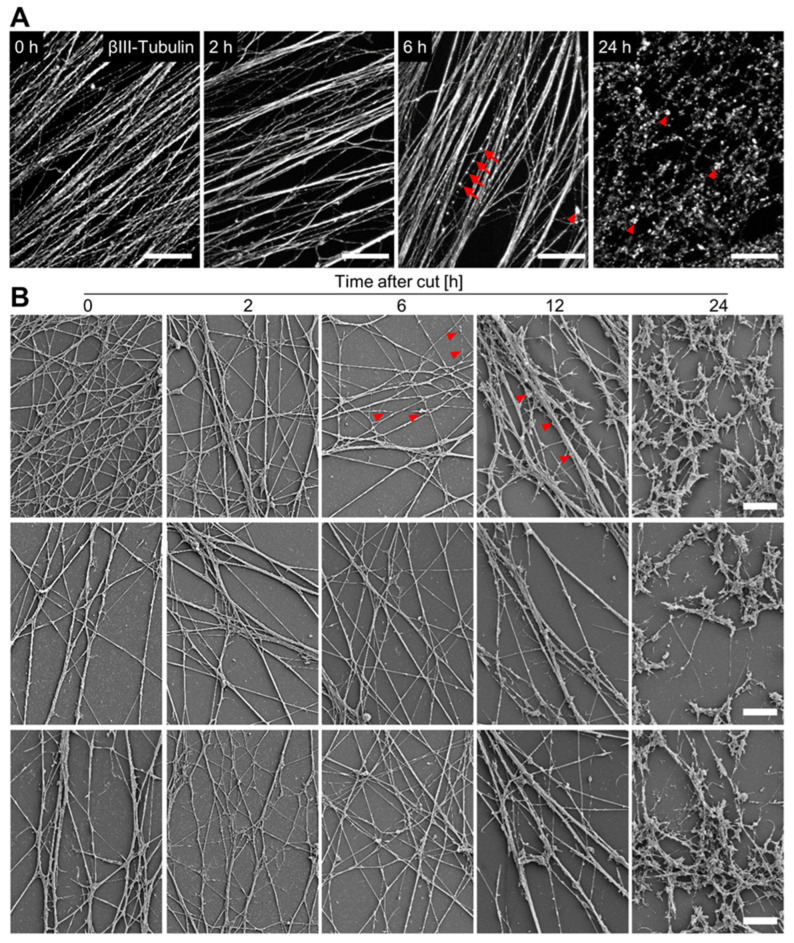
Morphological features of axotomy-induced neurite degeneration. (**A**) NOC were produced as described in Figure 2. They were fixed at different time points after axotomy and immunostained against βIII-tubulin. Images were recorded using epifluorescence microscopy. Arrows indicate neurite beading, and arrowheads indicate tubulin aggregates in fully fragmented neurites. Scale bar = 50 µm. (**B**) NOC were isolated and fixed at different time points after axotomy. Scanning electron microscopy images were recorded at 2500× magnification. Three representative images per time point are shown. Red arrowheads indicate exemplary neurite fragmentation. Scale bars = 10 µm.

A major application of NOC may be the study of axotomy-induced neurite degeneration (AIND). As an important basis for such research, we investigated how long the NOC would stay structurally intact, and in which way the degeneration proceeded. We fixed NOC at different time points after the removal of the cell bodies and stained microtubules with antibodies against βIII-tubulin. In healthy neurites, the microtubule cytoskeleton is well-aligned with the main neurite tracks and has smooth, sharply delineated features. This morphology remained intact for up to 6 h after axotomy. After this time, the microtubules looked less smooth, and they broke into structures resembling beads on a chain. This clearly indicated the beginning of neurite fragmentation. At 24 h after axotomy, all microtubules showed severe fragmentation throughout the whole culture (Figure 4A).

To investigate whether microtubule fragmentation was driven by neurite fragmentation, we performed SEM imaging of neurites fixed at different time points after axotomy. We observed the first signs of fragmentation at 6 h after axotomy. This correlated well with first signs of microtubule breakdown. At 12 h after axotomy, neurites were severely fragmented and showed extensive beading. After 24 h, neurites were completely fragmented (Figure 4B).

As this sequence correlated with the observations on microtubule integrity, we suggest that microtubules appeared increasingly fragmented because neurites were degenerating. Vice versa, these data suggest that immunostaining for microtubules can be used as a readout for neurite degeneration in the NOC model.

To test whether neurites remained viable after axotomy, we stained cut neurites at different time points after axotomy with the live cell dye calcein-AM. Calcein-AM can enter cells, where it is cleaved into the fluorescent molecule calcein, which is not able to cross cell membranes. Positive calcein staining therefore indicates the integrity of cell membranes around a whole cell or around any cell fragment enclosed by a plasma membrane. Thus, the major endpoint upon calcein staining is not the total fluorescence, but the fine structure of a detailed fluorescent image. This shows, e.g., intact (longer, smooth) neurites, or neurites with signs of degeneration (membrane-enclosed blebs or the fragmentation of membrane-enclosed beads).

**Figure 5:**
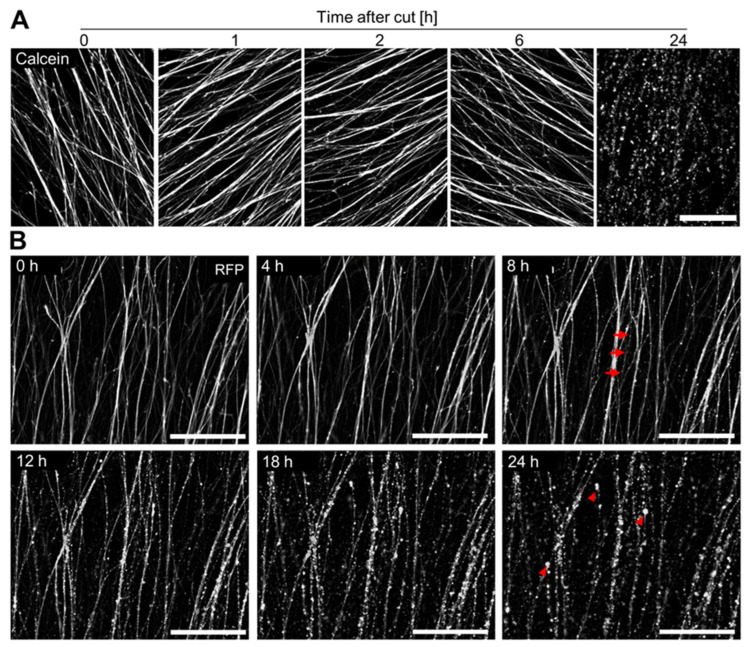
Live cell observation of progressive degeneration of NOC over time. (**A**) NOC were produced as described in Figure 2 and live-stained with calcein-AM at different time points after axotomy. Images were recorded by epifluorescence microscopy. Note that all stained areas (including fragments) are surrounded by “intact” plasma membranes (impermeable to calcein). Scale bar = 200 µm. (**B**) RFP-expressing LUHMES were used to track the neurite morphology in live NOC over time. Timelapse images were recorded every 10 min by epifluorescence microscopy. Representative images are shown (Supplementary Material Video S1). Arrows indicate neurite beading, and arrowheads indicate fully fragmented neurites. Scale bar = 200 µm.

Consistent with microtubule staining and SEM imaging, calcein staining confirmed that neurites stay structurally intact for up to 6 h after axotomy. Degenerated neurites (24 h after axotomy) often stained intensively with calcein. This indicated that the cell membrane of the neurite fragments stayed intact for extended periods (Figure 5A). As cell membrane integrity is the hallmark of certain forms of regulated cell death (e.g., apoptosis [61]), but not of others (e.g., ferroptosis [62] or necrosis [61]), these data hint towards a regulated form of ND that maintains membrane integrity during the fragmentation process. This experiment also showed that calcein imaging could be used as an endpoint to evaluate the degree neurite fragmentation.

To track the same neurites over time after axotomy, we performed time series imaging with red fluorescent protein (RFP)-expressing LUHMES (Figure 5B, Supplementary Video S1). This allowed for the imaging of given NOC over time. With this approach, the first signs of degeneration became visible between 6 and 8 h after axotomy, while neurites were >90% degenerated at 18 h after axotomy. Consistent with calcein staining, the maintenance of cytosolic RFP levels during degeneration also indicated the maintenance of membrane integrity. This method allowed the observation of neurite degeneration at a high temporal resolution and might be especially useful when interventions intended to delay the degeneration of neurites are of interest.

### 3.5. Quantification of Neurite Fragmentation

**Figure 6:**
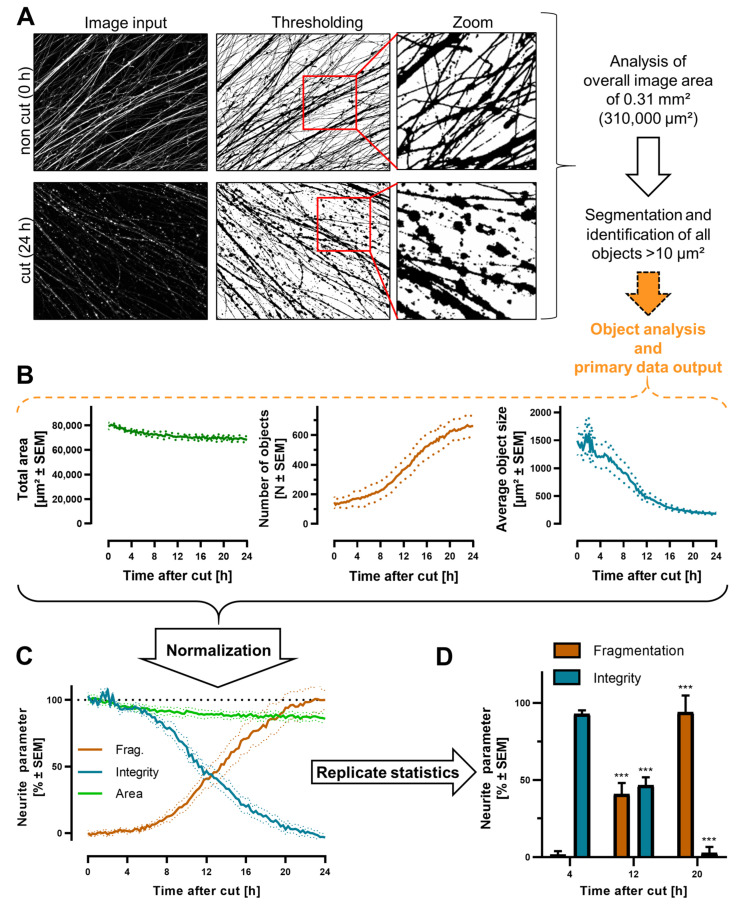
Workflow for quantification of neurite degeneration in NOC. (**A**) For quantification of neurite degeneration in NOC, fluorescence images of RFP were recorded at different time points after the axotomy (c.f. Figure 5B). Images were background corrected and then converted to binary (black–white) images by thresholding. Note that the same algorithm was also applicable to calcein-stained NOC. All objects >10 µm^2^ were identified in each image and used for further analysis. (**B**) Object data were quantified and the raw data output was plotted over time. This included the total object area (= neurite-covered area), the number of objects per image (providing information on the fragmentation level), and the average size of the objects identified per image (providing information on neurite integrity). (**C**) The raw data were normalized for standardization across experimental runs. The number of objects was normalized to the absolute numbers of fully degenerated cultures. Images of non-cut neurites were regarded as 0% fragmented. The average size of objects was normalized to absolute numbers of an intact control. (**D**) Normalized values were used to replicate statistics at individual time points. To evaluate statistical significance, a one-way ANOVA was performed, with Dunnet’s post hoc test, relative to 0 h. *** *p* < 0.001. In the example given, five cultures were analyzed.

The SEM and other approaches described the time course of neurite degeneration, but there was a need for a more quantitative readout to compare between culture dishes, experimental runs, or even experiments with changed culture parameters. To this end, we developed an observer-independent automated image analysis algorithm. It was based on sets of images captured from either RFP-expressing NOC or calcein-stained NOC after axotomy (Figure 6A). These images were processed to obtain binary images for further analysis. To quantify the morphological changes in the neurites, we analyzed the total neurite-covered area in the images over time, as well as the average size of objects and the count of all objects in the images (Figure 6B). The neurite-covered area did not change drastically within 24 h, as fragments formed from intact neurites were still fluorescent (see above). Therefore, data on the neurite-covered area were used as background controls for “valid” culture wells. In a typical axotomy experiment, the neurite-covered area was not reduced lower than 70% at 24 h after axotomy. It is thus suggested only to evaluate NOC with >70% “neurite-covered area”, compared to intact high quality control NOC, directly after axotomy. After the cut, the count of detected objects increased because the fragmentation of neurites led to more objects per image. Conversely, the average size of objects decreased because the detected fragments were smaller than intact neurites. Based on these principles, the image analysis algorithm derived parameters “fragmentation” (from the count of particles) and “integrity” (from the average size of particles) (Figure 6C).

As an exemplary data set, we selected images of RFP-expressing NOC taken at specific time points after axotomy. As several NOC were used for this experiment, a statistical evaluation of the neurite parameters was possible (Figure 6D). These quantitative data on degeneration over time reflected the qualitative observer-based assessment of ND. They were used as observer-independent endpoints for all following experiments.

### 3.6. Pharmacological Intervention against Neurite Degeneration

**Figure 7:**
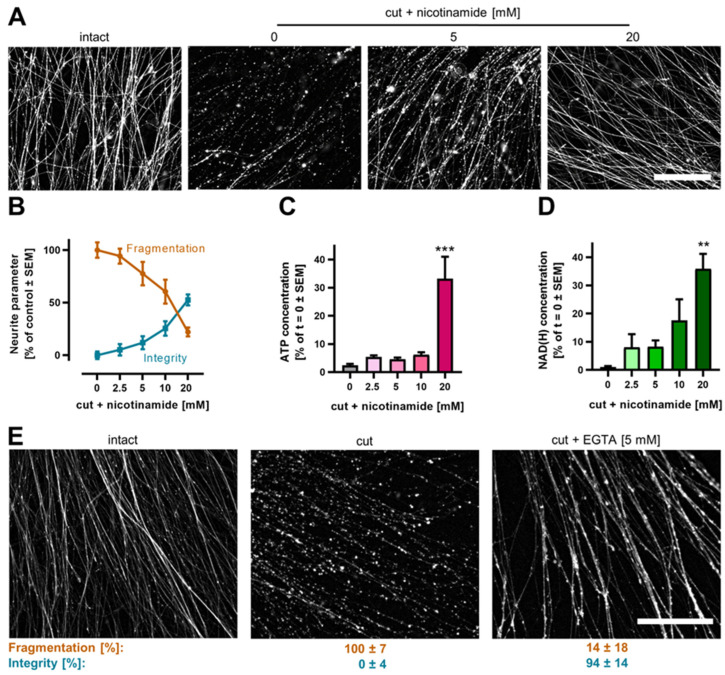
Modulation of neurite degeneration by pharmacological interventions. (**A**) LUHMES 2.5D cultures (DoD15) were generated, as in Figure 2. They were treated with different concentrations of nicotinamide 15 min before axotomy. At 18 h after axotomy, neurites were stained with calcein-AM and imaged by epifluorescence microscopy. Representative images are shown. Scale bar = 200 µm. (**B**) Quantification of neurite fragmentation and integrity 18 h (as in Figure 6) after axotomy, in the presence of different concentrations of nicotinamide. (**C**) ATP concentration in neurites 18 h after axotomy, in the presence of different concentrations of nicotinamide. Data were normalized to samples from NOC prepared immediately after axotomy. (**D**) Total concentrations of reduced plus oxidized nicotinamide adenine dinucleotide (NAD(H)) in neurites 18 h after axotomy, in the presence of different concentrations of nicotinamide. Data were normalized to samples from NOC prepared immediately after axotomy. A one-way ANOVA was performed, followed by Dunnet’s post hoc test. ** *p* < 0.01. *** *p* < 0.001. (**E**) Cultures were treated with the calcium chelator EGTA (5 mM) 15 min before axotomy. At 18 h after axotomy, NOC were stained with calcein-AM and imaged using epifluorescence microscopy. Representative images are shown. Below the images, the quantitative data on fragmentation and integrity (±95% confidence interval) are given (N = 3, n = 5). Scale bar = 200 µm.

An important future application of NOC is the testing of modulatory mechanisms and agents. To exemplify the suitability of the model for this purpose, we tested whether interventions known to be successful in other models would protect neurites from degeneration. In a first approach, nicotinamide (Nam) was selected, as it has been repeatedly shown to delay AIND [42,63,64]. In NOC, Nam slowed axotomy-induced degeneration in a concentration-dependent manner (Figure 7A). For quantification, we selected a fixed time point of 18 h after axotomy. NOC treated with 20 mM Nam showed less fragmentation compared to an untreated control, while the integrity was greatly increased (Figure 7B). To test if the improved structural integrity of neurites treated with Nam was accompanied by improvements in other measures of viability, we determined the ATP concentration of NOC. Untreated NOC lost >95% of the initial ATP concentration. Neurites treated with 20 mM Nam still had about one third of the ATP levels at 18 h after axotomy, compared with freshly isolated NOC (Figure 7C). To contextualize these relative data, we extracted information about absolute ATP levels from the literature. Several studies suggest the ATP concentration within neurons to be in the 2–3 mM range [65,66,67,68]. This value is also consistent with highly detailed metabolite quantifications in other cells [69]. Under the assumption that a similar concentration range is also present in LUHMES NOC, NOC rescued by Nam would still contain ATP in the several hundred µM (specifically, around 700 µM) range. This concentration would easily allow for most biochemical processes; it would also be sufficient for the survival of neurons [70,71,72]. The continued presence of ATP after 18 h (normally dissipated within minutes in neurons upon block of production [73]) also indicates continued ATP production.

In a second approach, we measured how nicotinamide adenosine dinucleotide (NAD^+^) levels would be affected by axotomy, and how this was modulated by the addition of Nam. While NAD^+^ was completely depleted 18 h after axotomy, NOC treated with Nam maintained up to ca. 35% of their NAD^+^ (Figure 7D). NAD maintenance is known to be absolutely essential for the preservation of neurite integrity [15,74,75,76,77], and the depletion of the dinucleotide is known to be central in neurodegeneration. Two important NAD^+^-depleting enzymes activated upon neurite/neuronal damage are SARM1 and PARP [22,78,79,80]. Two likely mechanisms for the protective role of Nam are (i) feedback inhibition of SARM1 activity and (ii) increasing basal NAD^+^ levels (which then prevent SARM1 activation), but more mechanistic studies are needed.

After this first proof-of-concept for a metabolically controlled degeneration process in NOC, we explored another (mechanistically entirely different) intervention known to protect against AIND: the prevention of an increase in free intracellular Ca^2+^ levels [24,81,82]. NOC, treated with the Ca^2+^ chelator ethylene glycol-bis(β-aminoethyl ether)-N,N,N′,N′-tetraacetic acid (EGTA), showed a decreased neurite fragmentation and an increased neurite integrity 18 h after axotomy (Figure 7E). This data set demonstrated again that AIND of NOC can be strongly modified by metabolic interventions. As a third type of intervention, we considered some processes suggested to be involved in the destruction of neurite structural elements. For instance, the protease family of calpains has been found to have such a role in some models [83,84]. We therefore explored the potential protective effect of calpains inhibitors that we had found previously to attenuate excitotoxic cell death [85,86]. However, we did not observe a significant reduction or slowing of neurite degeneration (Figure S4). This is well in line with clinical findings that failed to show the efficacy of calpain inhibitors over the past 20 years [87]. In summary, we have shown here that degeneration of NOC can be prevented by some, but not all, experimental approaches. Thus, NOC are a CNS neuron-based model that may be used to test more approaches like these. The easy biochemical accessibility would then also allow mechanistic follow-up studies.

## 4. Conclusions and Outlook

In this study, we developed a method to generate cultures of isolated human neurites. This system allowed for not only the selective imaging of neurites but also the biochemical analysis of the neurite fraction. We provided proof-of-concept that protein, RNA, and metabolites can be quantified in NOC. Moreover, such data may be put in context with data on structural protection, as exemplified here for Nam.

The possibility of analyzing neurites (morphologically and biochemically) will be useful for future studies of cellular processes regulating neurite maintenance, such as axonal transport or the regulation of local protein levels [59]. For instance, there are still open questions regarding the maintenance of axonal mitochondria or the supply of the neurite with short half-life proteins [88]. Our system might offer an adequate platform to investigate these issues in human neurons. To study these intricate cellular processes in live neurites, genetic reporters could be used to examine various neurite functions. The local and real-time measurement of, e.g., Ca^2+^ [24], NAD^+^ [89], or ATP [90,91] levels would lead to a better understanding of neurite death.

In an initial analysis, we demonstrated the feasibility of mRNA analysis in the neurite fraction. The next step would be the analysis of the whole neurite transcriptome. At present, there is a dearth of such data [92], and we are not aware of any such information on midbrain neurons. As NOC allow for the purification of relatively high amounts of neurite RNA, the generation of RNAseq data appears feasible.

Another potential use of NOC is the study of AIND. We provided here already detailed data on disintegration after axotomy. The quantification algorithm developed by us allows the capturing of objective observer-independent data. Our example intervention studies suggest a potential use of NOC as a platform to study the mechanism of AIND. A specific application may be the degeneration observed in neural transplants [93]. For instance, the anti-apoptotic gene bcl-2 [94] and caspase inhibitors [95] can stabilize neurites to some extent. The NOC model would allow for the identification of more potent or efficacious treatments, which would increase the effectiveness of dopaminergic neuron transplantations [96].

The NADase SARM1 was identified to be the main executor of programmed axon degeneration [22]. Most studies on this emerging pathway of programmed ND have been performed on rodent peripheral nervous system (PNS) neurons [46]. The system presented here would allow for the study of whether the same mechanism also triggers AIND in human CNS neurons. We could observe a protective effect of Nam, suggesting a central involvement of NAD^+^ metabolism in the AIND of NOC. An obvious next step is the study of the role of SARM1. The availability of a suitable human CNS neuron model would allow for the identification of cellular events downstream of SARM1 activation, and also the discovery of potential new interventions.

The 2.5D model (without axotomy) might also prove useful for toxicological studies. The specific toxicity of many compounds often depends on the neurite length [97]. A system with neurites of several mm lengths may allow for a higher sensitivity of detection [52,53,54]. It would also allow for the quantification of toxicant effects on axonal transport and other functional parameters.

The approach taken here may possibly be transferred to other human cell lines, or to human induced pluripotent stem cell (iPSC)-generated neurons. The choice of cell may depend on the exact model requirement. LUHMES have been found in some comparative studies [98,99] to be particularly well-suited to studying neuronal death.

For some research questions, the culture model would need to be developed further. For instance, the central cell body dome may be replated to test the potential regrowth of injured neurites. While CNS neurons normally fail to regrow neurites, PNS neurons can regenerate [100]. Reasons for the failure of CNS neurons to regenerate neurites remain largely unclear [101]. This also limits advances in drug development for, e.g., spinal cord injury. It appears, thus, attractive to modify the 2.5D cultures to allow for the investigation of CNS neuron regrowth.

Finally, further studies may make use of the fact that, here, we used human dopaminergic CNS neurons. On this basis, one may investigate a potential link between the processes driving AIND in this system and the triggers of ND degeneration that are suspected to occur in PD. The use of transgenic LUHMES lines expressing various PD-associated genes [57,102] would link these research fields. Possibly, such studies can then address questions, such as whether protein aggregates mimic axotomy, in the sense of impairing axonal transport.

## Data Availability

All data is available upon request.

## Supplementary Materials

The following supporting information can be downloaded at: https://www.mdpi.com/article/10.3390/cells13030242/s1, Figure S1: SEM images of plated spheroids covered by Matrigel; Figure S2: Details on the procedure and normal variability of NOC production; Figure S3: Quantification of relative RNA abundances in neurites; Figure S4: Failure of calpain inhibitors to rescue NOC from axotomy induced degeneration; Table S1: Primers used for real-time qPCR; Table S2: Antibodies used in this study; Video S1: Progressive degeneration of RFP-expressing neurites after axotomy.
