# Supplementary material for: Preparation of Viable Human Neurites for Neurobiological and Neurodegeneration Studies
Source: Cells. 2024 Jan 27;13(3):242. doi: 10.3390/cells13030242 (PMC10854604; doi:10.3390/cells13030242)
Supplement: Supplementary file 1 [file cells-13-00242-s001.zip › Br乴l2024_Supplementary_Material_revision.pdf]

Supplementary information for

Preparation of viable human neurites for neurobiological and neurodegeneration studies

Markus Brüll, Nils Geese, Ivana Celardo, Michael Laumann and Marcel Leist

Table of Contents

Figure S1	SEM images of plated spheroids covered by Matrigel	page 2
Figure S2	Details on the procedure and normal variability of NOC production	page 3f
Figure S3	Quantification of relative RNA abundances in neurites	page 5
Figure S4	Failure of calpain inhibitors to rescue NOC from axotomy induced degeneration	page 6
Table S1	Primers used for real-time qPCR	page 7
Table S2	Antibodies used in this study	page 8

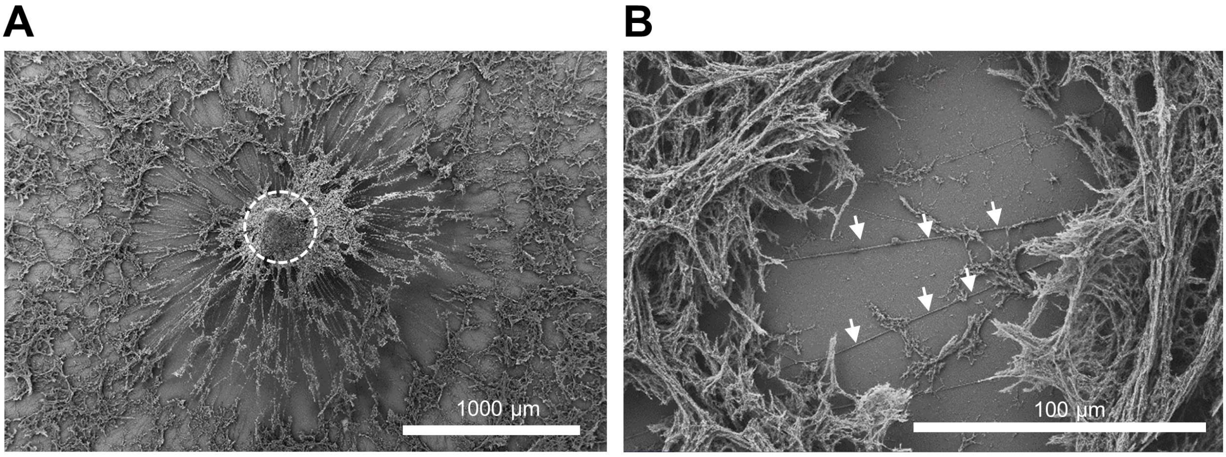

Supplementary Figure S1: SEM images of plated spheroids covered by Matrigel

A: SEM image of an intact 2.5D spheroid covered by Matrigel. The spheroid is representative of the standard procedure to produce NOC, as described in fig. 1. It was generated and plated into a 1:40 Matrigel suspension as described in fig. 1. Note that for better neurite display (e.g. fig. 3) the Matrigel amount was reduced (minimal Matrigel coating by removing suspension before plating). The dotted circle indicates the central spheroid. **B:** Detail image of Matrigel-covered surface. White arrows indicate neurites growing below the Matrigel layer.

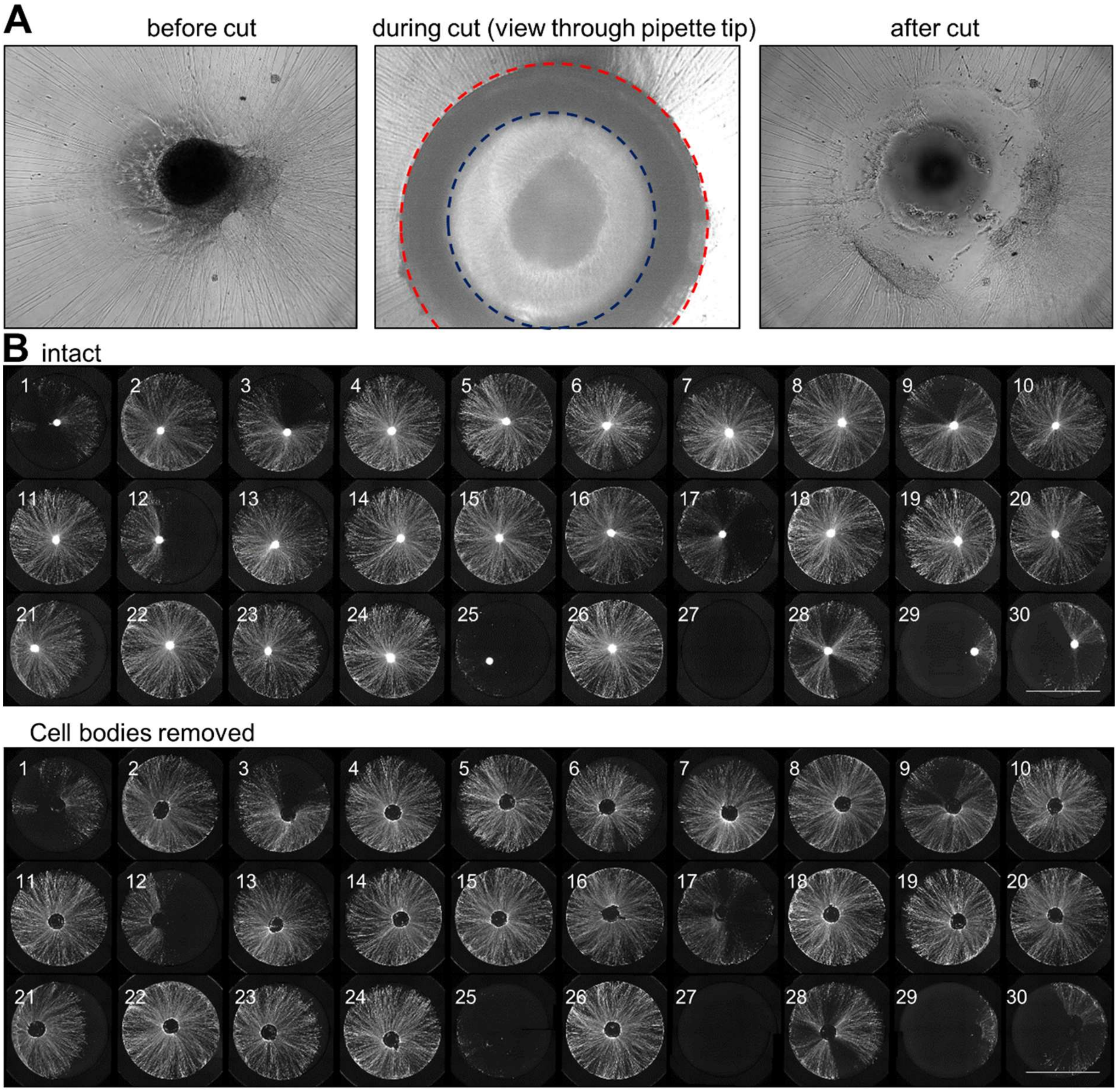

C

n = 30	**accepted**	**Not accepted**		
**Neurite quality**	good neurites	Partially absent	Fully absent	empty
**Score (n)**<br>**Abundance (%)**	20<br>(67%)	7<br>(23%)	2<br>(7%)	1<br>(3%)
**Example well**	Well #4	Well #17	Well #25	Well #27

Supplementary Figure S2: Details on the procedure and normal variability of NOC production

A: Phase contrast images of 2.5D spheroids before, during and after axotomy and cell body removal. The red dotted circle indicates the outer border of the pipette tip, the blue dotted circle indicates the inner lumen. The smooth grey ring in between is the plastic wall of the pipette tip used for cutting. Note in the right image, that immediately after the cutting there is neurite contraction and neurite debris at the inner cut site. **B:** Display 30 exemplary wells of plated spheroids at 5 days after plating. Upper images are before the cut; lower images are after cell body removal. Spheroids were stained with calcein-AM and whole wells of a 96 well plate were imaged by epifluorescence microscopy. For each micrograph displayed, 12 microscope images (3x4) were stitched together per well. Scale bar = 5 mm. **C:** For subsequent analysis, only spheroids which showed full radial neurite outgrowth were used. Here, the analysis of the quality control of the 30 wells shown in B is given as a representative example.

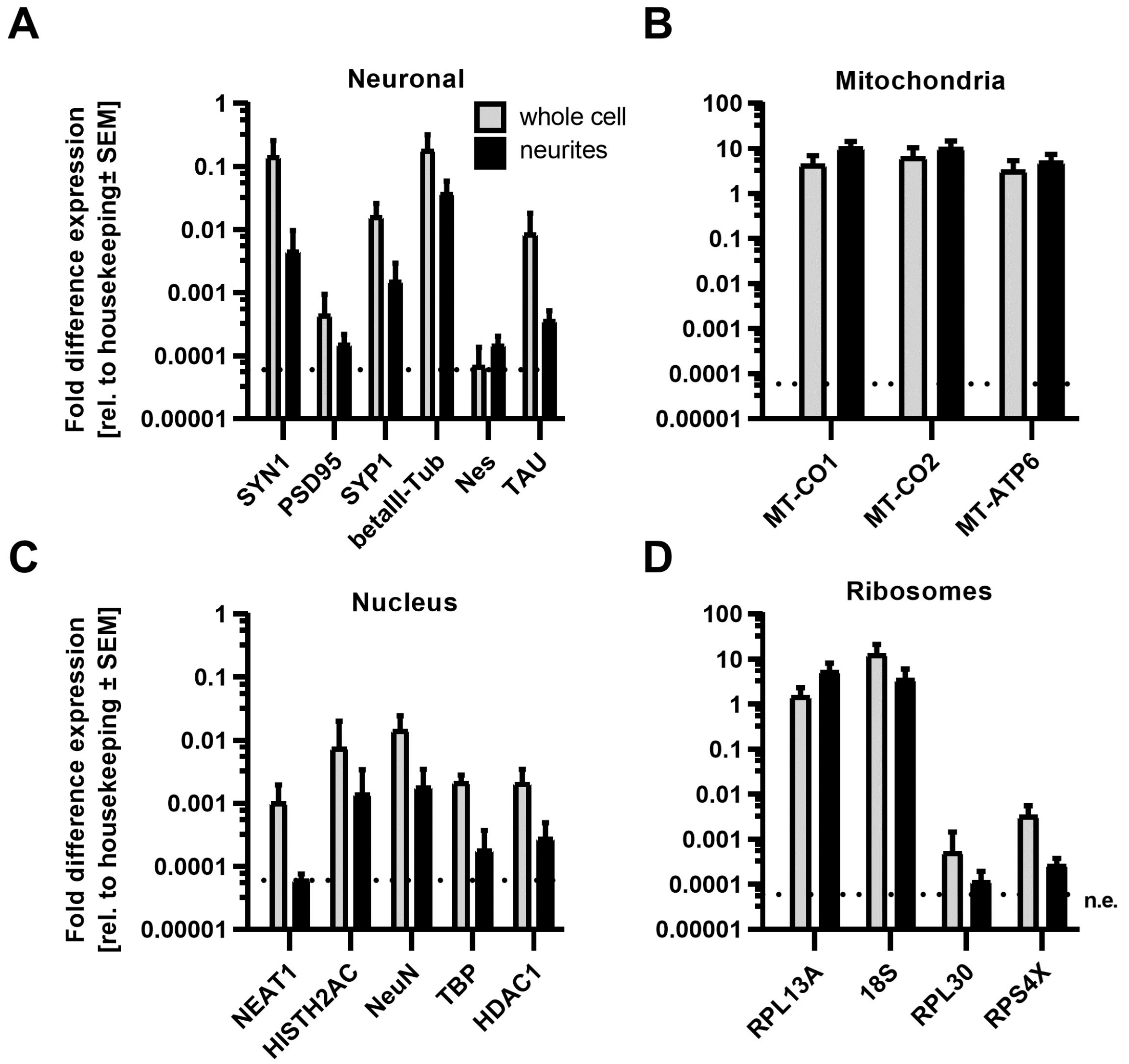

Supplementary Figure S3: Quantification of relative RNA abundances in neurites

RNA was isolated from NOC or whole cell preparations. The same amount of total RNA was used for anaylsis by real-time quantitative PCR (RT-qPCR). Expression data was normalized to the geometric mean of ACTB and RPL13A levels (housekeeping genes). Relative expression to housekeeping genes is shown. Genes which were not detected after cycle 40 were regarded as not expressed (n.e.). **A:** Neuronal marker genes. **B:** Genes expressed by mitochondria. **C:** Genes of proteins typically found in the nucleus. **D:** Genes encoding for ribosomal subunit proteins or ribosomal RNA.

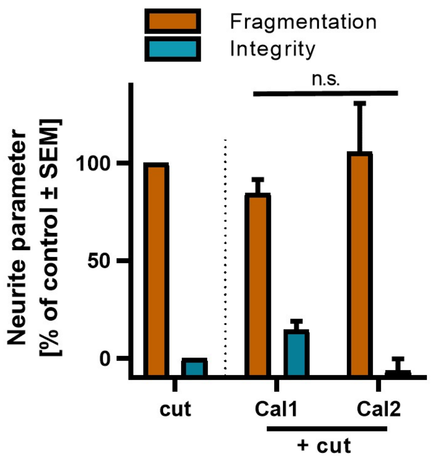

Supplementary Figure S4: Failure of calpain inhibitors to rescue NOC from axotomy induced degeneration

Cultures were treated with the calpain inhibitors 1 (Cal1) and 2 (Cal2) before axotomy. 18 h after axotomy, neurites were stained with calcein-AM and imaged by epifluorescence microscopy. Quantification of neurite fragmentation and integrity 18 h after axotomy is shown.

Supplementary table S1: Primers used for real-time qPCR

target transcript	sequence forward primer	Tm [°C]	sequence reverse primer	Tm [°C]	product size [bp]
ACTB	GCACAGAGCCTCGCCTT	59.7	GTTGTCGACGACGAGCG	58.6	93
RPL13A	GGTATGCTGCCCCACAAAACC	62.1	CTGTCACTGCCTGGTACTTCCA	61.9	205
GAPDH	ATGGAGAAGGCTGGGGCTCA	63.1	AGTGATGGCATGGACTGTGGTCAT	64.2	234
MT-CO1	TACGTTGTAGCCCACTTCCAC	60.0	GAGTAACGTCGGGGCATTCC	60.8	209
MT-CO2	CCTCATCGCCCTCCCATCCC	64.3	TCAAGGAGTCGCAGGTCGCC	64.6	189
MT-ATP6	TTCAACCAATAGCCCTGGCC	60.3	GTAGGCTTGGATTAAGGCGAC	58.8	185
18S	GACTCAACACGGGAAACCTCACC	63.5	TCTAAGAAGTTGGGGGACGCCG	64.3	204
DLG4	CACTCCTCACAGTGCTGCAT	60.3	TGTCTTCATCTTGGTAGCGG	57.3	111
PARK7	AAGGGCCTGATAGCCGCCAT	63.9	GCGCAAACTCGAAGCTGGTC	62.5	202
RPL30	TGAAGAGCTTTGCATTGTGG	56.9	CGGAGTTACAAATGGCAACC	57.4	137
HDAC1	CACCCGGAGGAAAGTCTGT	58.9	GGGCGATAGATTTCCATTTTT	54.6	150
SYP1	GGTGGCTGGGGGTCAGTTCC	64.9	TTGGTGCAGCCTGAAGGGGT	64.2	199
TBP	GGGCACCACTCCACTGTATC	60.1	GCAGCAAACCGCTTGGGATTATATTCG	65.1	198
TUBB3	AACTACGTGGGCGACTCGGA	63.4	GTTGTTGCCGGCCCCACTCT	65.5	198
RPS4X	TGCAGCAGTTAGGGAACCCATG	62.9	CACCTTGATGAGGGGATCGGGG	64.4	198
TAU	CCATGCCAGACCTGAAGAAT	57.6	CACACTTGGACTGGACGTTG	59.1	129
RBFOX3	AGCCCGGGAGAAGCTGAATG	62.3	GTGCCGGTGGTGGGGTAGGG	67.1	195
SYN1	TCAGACCTTCTACCCCAATCA	57.8	GTCCTGGAAGTCATGCTGGT	59.7	127
NEAT1	GCCCATGAATGCCAGCAGGC	64.8	GGCTCCATCTGCAAGCTCCA	62.6	198
HIST1H2AC	CAACTACGCAGAGCGGGTTG	61.6	TGAGCAATGGTCACCCGGCC	65.3	201

Supplementary table S2: Antibodies used in this study

**target**	**species**	**stock concentration**	**dilution**	**supplier**	**catalog number**
α-Tubulin	rabbit	8 µg/ml	1:5000	Cell Signaling	2125
β-Actin	mouse	2.0 - 2.5 mg/ml	1:5000	Sigma-Aldrich	A5228
Tom20	rabbit	200 µg/ml	1:1000	SantaCruz	sc-11415
Histone H3	rabbit	9 µg/ml	1:1000	Cell Signalling	9715S
βIII-Tubulin	mouse	1.0 mg/ml	1:500	BioLegend	801202
Anti-mouse-IgG-HRP	goat	0.8 mg/ml	1:10 000	Jackson	115-036-068
Anti-rabbit-IgG-HRP	goat	1.0 mg/ml	1:10 000	Biozol	VEC-PI-1000
Anti-mouse-IgG2a-Alexa-647	goat	2 mg/ml	1:1000	Invitrogen	21241
